# Glove single-port laparoscopy-assisted transanal total mesorectal excision for low rectal cancer: a preliminary report

**DOI:** 10.1186/s12957-019-1744-z

**Published:** 2019-11-30

**Authors:** Wanglin Li, Boye Dong, Baifu Peng, Jiabao Lu, Zixin Wu, Guanwei Li, Jie Cao

**Affiliations:** 1 Department of Colorectal Surgery, Guangzhou First People’s Hospital, School of Medicine, South China University of Technology, No.1 Panfu Road, Guangzhou, 510180 Guangdong China; 2 Department of Colorectal Surgery, Guangzhou First People’s Hospital, Guangzhou Medical University, School of Medicine, Guangzhou, Guangdong China; 3 Department of Gastrointestinal Surgery, Shunde Hospital of Southern Medical University, Shunde, Foshan, China

**Keywords:** Glove port, Single-port laparoscopic surgery, Transanal total mesorectal excision, Rectal cancer

## Abstract

**Purpose:**

Glove single-port laparoscopy-assisted transanal total mesorectal excision (TaTME) has been successfully carried out in our medical center. The purpose of this study is to evaluate the feasibility of this emerging operation.

**Methods:**

This technique was performed by self-made glove single-port laparoscopic platform to radically resect low rectal cancer. Short-term postoperative results, including complications, length of hospital stay, and follow-up results were collected and analyzed statistically.

**Results:**

There are five consecutive patients (three males, two females) who underwent this surgery and included in this study. The mean distance from the tumor to the anal verge was 4.8 cm (range 4.0–6.0). The surgery was completed in all cases, and the rectal tumor was removed successfully without conversion; circumferential margins of all the excised specimens were negative. The mean time of operation was 338.00 min (range 280–400). The average number of lymph node dissection was 12.20. The average postoperative hospital stay was 8.60 days. During the follow-up (14.80 ± 1.92 months), all preventive ileostomies were successfully closed in about 3 months after the surgery, all patients had satisfactory anal function, and no tumor recurrence was found.

**Conclusion:**

Glove single-port laparoscopy-assisted TaTME has a significant effect in specific patients with low rectal cancer, with rapid recovery and high safety. Prospective randomized studies involving more case counts and long-term follow-up results, especially oncologic outcomes, are needed to validate this technique.

## Introduction

In recent decades, with the application and maturity of laparoscopy even robotics, minimally invasive surgery has developed rapidly in the field of colorectal cancer, and many new technologies have come into being. In recent years, the concept of natural orifice transluminal surgery (NOTES) has attracted the attention of surgeons. The “incision-free” concept of NOTES combined with the operational skills of laparoscopy shows perfect minimally invasive effect, as well as good safety and operability. Compared to conventional laparoscopic surgery, NOTES provides many potential advantages for selected rectal cancer patients, such as avoiding transabdominal incisions and their related complications [1].

Transanal total mesorectal excision (TaTME) is an emerging NOTES surgery. Previously, most of the reported TaTME cases were performed through traditional multi-port laparoscopic surgery (MPLS). However, as a developed technique, single-port laparoscopic surgery (SPLS)-assisted TaTME was reported relatively fewer, though it is becoming more and more noticeable in recent years [2–4]. With great interest in SPLS, we successfully performed TaTME with self-made glove single-port laparoscopic platform in our medical center. According to the statistics, the expense of this self-made platform is about 2500 dollars lower than that of the single-item platform, such as GelPoint platform. Now, we introduce the preliminary outcomes on patients who underwent TaTME using this self-made glove single-port laparoscopic platform, including the surgical platform and operation details, short-term outcomes, and follow-up results.

## Materials and methods

This study started in January 2015. Specimens were assessed according to our protocol. Informed consent was obtained after detailed explanations of the benefits, possible complications or risks, and alternatives of the operation.

### Patient

Patients with histologically proven T1-T3 low rectal adenocarcinoma were included in the study. All tumors were in a distance less than 6 cm from the anal verge. Patients with local recurrence and distant metastases, locally advanced tumors (cT4 stage), acute intestinal obstruction, poor anal function, history of ulcerative colitis or Crohn’s disease, and familial adenomatous polyposis were excluded. All patients underwent colonoscopy, lower abdominal magnetic resonance imaging (MRI), thoracoabdominal computed tomography (CT) scan, and sphincter manometry for adequate preoperative evaluation. According to the guidelines for the diagnosis and treatment of colorectal cancer, patients with clinically positive lymph node received neoadjuvant chemoradiotherapy.

### Surgical technique

Patients who underwent neoadjuvant therapy will wait 8 to 12 weeks since completing radiotherapy before surgery. All patients received mechanical bowel preparation (MBP) combined with oral antibiotics for preoperative bowel preparation. MBP was done using oral polyethylene glycol electrolyte powder or cleaning enema. Oral antibiotics commonly used are ciprofloxacin and metronidazole.

The anesthesia method was general anesthesia with endotracheal intubation. After successful anesthesia, the lithotomy position was taken, and the head was lowered, the lower limbs were raised and outspread, fully exposing the anus. To achieve fast recovery after surgery, the stomach tube was not inserted if it was not necessary, and the urinary catheter was pulled out within 24 h after surgery. A drainage tube was placed to drain the pelvic cavity accumulating liquid.

**Fig. 1:**
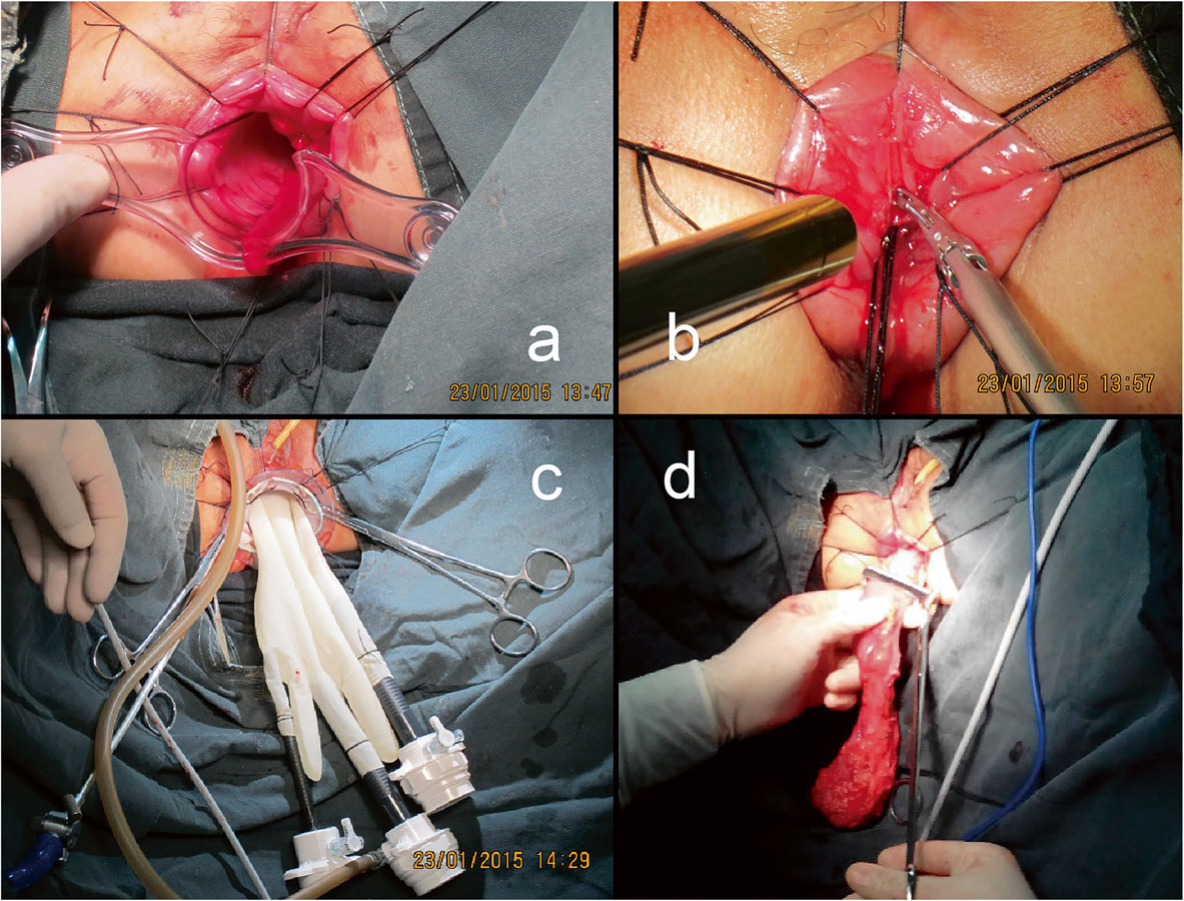
Surgical approach. **a** The rectum was fully exposed with an anal retractor, then the tumor lesion location was confirmed and the level of incision in the lumen was accurately decided. **b** The rectal lumen was tightly occluded, and a full-thickness circumferential dissection was performed. **c** The glove single-port platform was constructed by the surgical gloves and trocars. **d** The specimen was extracted through the anus

Key surgical steps were as follows: Firstly, we rinsed the operative region including the rectum lumen with sterile solution and fully exposed the rectum with an anal retractor, then confirmed the tumor lesion location and accurately decided the level of incision in the lumen (Fig. 1a). Secondly, we used purse-string suture to close the rectum tightly, then performed a full-thickness circumferential dissection (partial intersphincteric dissection was needed for ultra-low cancer) (Fig. 1b). Thirdly, the self-made glove single-port platform was constructed and inserted. The platform was structured mainly using surgical gloves and wound protectors. The surgical glove fingertips were cut, and then, trocars (one 10 mm, one or two 5 mm) were inserted through the tip-holes and secured. Then, the operating instruments and endoscope lens were inserted through the trocars, and the instruments were fixed on the fingertip holes of the glove with stitches. In this process, attention should be paid to avoid the “chopstick effect” of operating instruments and to ensure that trocars are well fixed with the fingertip holes of the glove to avoid air leakage. Since the anal canal anatomy would not be suitable for setting a wound protector, an anal speculum was used instead to construct the glove anal single-port laparoscopic platform. The anal speculum was fixed on the perianal skin of the patient, and then, the glove operating platform was securely fixed to the anal speculum with sutures. Then, the glove single-port platform was finished and the pneumo-pelvis was established (Fig. 1c). Dissection was performed first on the posterior side, then the lateral side, paying attention to the protection of the ureter and pelvic plexus. Finally, the anterior side of the rectum was dissected along the Denonvilliers’ fascia. The abdominal cavity was accessed by incising anteriorly or through the right side.

In the abdominal approach, the glove single-port platform was placed at the future ileostomy site. After abdominal exploration, if abdominal dissection was difficult, an additional umbilical port was added, and then adopted the medial-to-lateral approach: firstly, we used the ultrasonic scalpel and absorbable clips to separate, skeletonize, ligate, and divide the submesenteric blood vessels, as well as dissection of the regional lymph nodes. And then, we dissected the descending colon, sigmoid colon, and the upper rectum until the tumor was in free continuity with the previous transanal operation.

Finally, we pulled the specimen out through the anus (Fig. 1d). Then, we used the stapler to perform an end-to-end coloanal anastomosis and created a protective ileostomy to ensure better healing of the anastomosis. The rubber tube was routinely left for 3–5 days as a pelvic drainage.

## Results

From January to June 2015, there are five consecutive patients (including three males and two females) who were included in this study. The average age was 59.4 years (range 43–68). Body mass index (BMI) ranged from 19.5 to 25.2. All lesions were proved to be adenocarcinoma located in low rectum by taking pre-operative biopsies, with mean distance from the anal verge of 4.8 cm (range 4–6). The demographic characteristics, operative information, pathologic outcomes, and follow-up data are summarized in Table 1.

**Table 1:** Outcomes of glove single-port laparoscopy-assisted transanal total mesorectal excision

	Patient#1	Patient#2	Patient#3	Patient#4	Patient#5	Mean ± SD
Age (years)	43	58	68	62	66	59.4 ± 9.94
Gender	Female	Male	Male	Female	Male	–
Body mass index (kg/m^2^)	19.5	23.5	23.2	25.2	24.6	23.2 ± 2.22
ASA score	1	1	2	2	1	–
Underlying disease	No	No	HBP	No	No	–
Previous abdominal operation	No	No	No	No	No	–
Distance from anal verge (cm)	5.0	6.0	4.0	5.0	4.0	4.8 ± 0.84
Diameter of tumor (cm)	1.5	3.0	2.0	2.5	2.0	2.2 ± 0.57
Tumor position	Left lateral	Anterior	Posterior	Anterior	Anterior	–
Neoadjuvant therapy	Yes	No	No	No	No	–
Operative time (min)	280	360	400	310	340	338.0 ± 46.04
Estimated blood loss (ml)	50	150	50	80	50	76.0 ± 43.46
Length of specimen (cm)	9.0	10.0	8.0	9.0	10.0	9.2 ± 0.84
Lymph nodes harvested	12	13	11	12	13	12.2 ± 0.84
(y)TNM stage	yT1N0M0	T3N1M0	T1N0M0	T2N0M0	T2N1M0	–
Circumferential margin	Negative	Negative	Negative	Negative	Negative	–
Bowel movement (days)	3	2	3	4	3	3.0 ± 0.71
LOS (days)	8	7	9	10	9	8.6 ± 1.14
Conversion	No	No	No	No	No	–
Reoperation	No	No	No	No	No	–
Complications	No	Stoma prolapse	No	No	No	–
30-day readmission	No	No	No	No	No	–
Follow-up (months)	17.0	16.0	14.0	15.0	12.0	14.8 ± 1.92

*ASA* American Society of Anesthesiologists, *HBP* high blood pressure, *(y)* TNM stage for patients that received neoadjuvant chemoradiotherapy, *LOS* length of hospital stay

All cases underwent creation of protective ileostomies without conversions to open operation. The average operative time was 338 min (range 280–400). The mean estimated blood loss was 76 ml (range 50–150). In all specimens, the mesenteric fascia remained intact, and no tumor invasion was found at the distal and peripheral margins. The average number of lymph node dissection was 12.20 (range 11–13).

In terms of rehabilitation, the mean time for postoperative passing flatus was 3 days (range 2–4), indicating intestinal movement begins to recover; length of hospital stay ranged from 7 to 10 days with an average of 8.6 days. One patient experienced stoma prolapse slightly at 2 months after surgery. The patient was treated conservatively and made a fast recovery. Follow-up (14.8 ± 1.92 months, range 12–17) results showed that all ileostomies had been successfully closed; no tumor recurrence or fecal incontinence was found in the patients.

## Discussion

Developments in surgical techniques within each passing day have had improved patient outcomes, especially in the field of colorectal cancer surgery. To chase shorter wounds and faster recovery, new techniques such as SPLS have emerged [2, 5, 6]. Though SPLS have been shown to be feasible in colon cancer [7], SPLS for rectal cancer, especially for distal rectal cancer, is relatively more difficult and challenging [5, 7, 8].

The recently developed TaTME technique embodies the concept of NOTES [9] and may be a superior approach for rectal cancer [2, 10]. TaTME could resect the lesions completely and ensure negative circumferential margin, while it extracts the specimen through the anus and may avoid the vaginal or larger abdominal incisions [11].

According to the transanal endoscopic platform, TaTME can be classified as transanal endoscopic microsurgery-TME (TEM-TME) using TEM platform and transanal minimally invasive surgery-TME (TAMIS-TME) using TAMIS platform. Early on, at the time of TaTME implementation, the more common approach was TEM-TME [12]. In 2010, laparoscopy-assisted TEM-TME was reported by Sylla et al. and came out with no post-operative complications [11]. Along with the implementation and development of intraluminal minimally invasive surgery using the TAMIS platform, TAMIS was developed to achieve TaTME operation. Surgeons have now preferentially adopted the disposable multi-channels single-port platform for TAMIS [13, 14]. TaTME permits a clear and magnified filed to get access to the confined distal rectum from below [15]. Hence, it may reduce the difficulty of the operation and avoid some difficult situations encountered by conventional laparoscopic surgery, such as the use of stapling multiple times across the rectum, which increases the likelihood of anastomotic leak and involved distal resection margin [16].

However, when performing pure TaTME, it is challenging to divide the inferior mesenteric vessels and achieve colonic mobilization [12]. Many surgeons still tend to use the conventional MPLS as assistance because of the consideration of its flexibility and security. Recently, several groups have reported large case series of SPLS-TaTME [2, 3, 6] and pointed out that SPLS-TaTME approach might be easier to operate than the conventional MPLS approach. Reports of the SPLS-assisted technique also described using one or more additional trocars on a single-port basis for difficult dissection, such as a high splenic flexure [17, 18]. In the present study, we added one trocar in the umbilical position in one case. Since the umbilicus is the congenital scar, the post-operation scar at the umbilicus will still keep good cosmesis. We believe that it is a good choice to use this position if an additional port is required.

Because of the high equipment costs, low-income patients may hardly afford commercially available SILS-specific instruments and ports, such as GelPoint (Applied Medical Company). The glove port described in this study was constructed economically with a surgical glove, an anal speculum, and some trocars, saving a lot of equipment and platform costs.

To shorten the operative time, two teams could perform the first part and the second part of the presented surgery respectively at the same time. Due to the limitations of personnel and experience, we did not adopt this respective way during our study.

The major limitation of this study is the small sample size and selection bias. All patients were selected by the operating surgeon. Because of the short follow-up period, we could only draw limited conclusions about the oncological and short-term functional outcomes. The advantages of TaTME, especially for low rectal tumors or in the narrow male pelvis, demand this technique which continues to be refined and investigated.

## Conclusions

This study indicated that glove single-port laparoscopy-assisted TaTME has a significant effect in specific patients with low rectal cancer, with rapid recovery and high safety. Although the results showed that the short-term outcomes of this new technique were satisfactory, the sample size of this study was small; thus, prospective randomized studies involving more case counts and long-term follow-up results, especially oncologic and functional outcomes, are needed to validate this technique.

## Data Availability

All data generated or analyzed during this study are included in this published article and its supplementary information files.
